# Molecular characterization of rifampicin-resistant *Staphylococcus aureus* isolates in a Chinese teaching hospital from Anhui, China

**DOI:** 10.1186/1471-2180-12-240

**Published:** 2012-10-22

**Authors:** Wenjing Zhou, Wulin Shan, Xiaoling Ma, Wenjiao Chang, Xin Zhou, Huaiwei Lu, Yuanyuan Dai

**Affiliations:** 1 Department of Laboratory Medicine, Anhui Provincial Hospital, Hefei, China; 2 Anhui Medical University, Hefei, 230032, People’s Republic of China

**Keywords:** *Staphylococcus aureus*, MRSA, Rifampicin resistance, *rpoB* gene, MLST

## Abstract

**Background:**

*Staphylococcus aureus* (*S. aureus*) is a major nosocomial pathogen that causes a variety of infections and toxicoses. In recent years, the percentage of rifampicin-resistant *S. aureus* has increased rapidly in China. The aims of this study were to analyze 1) the level of rifampicin resistance in *S. aureus* and its correlation with mutations in the *rpoB* gene, and 2) the molecular characterization of rifampicin-resistant *S. aureus* isolates.

**Results:**

88 rifampicin-resistant *S. aureus* isolates were collected for this study. Of the 88 isolates, 83 (94.3%) were high-level rifampicin resistant (MIC≥8 mg/L) while the remaining 5 isolates (5.7%) had a low-level resistance to rifampicin (MIC, 2 to 4 mg/L). Four amino acid substitutions were found in the 88 isolates, which were 481His/Asn (95.5%), 466Leu/Ser (87.5%), 477Ala/Asp (6.8%) and 486Ser/Leu (4.5%) respectively. All mutations were found to be present in cluster I of the *rpoB* gene. The low-level resistant isolates were found to have only one mutation, while the high-level resistant isolates had at least two or more mutations. The most common multiple mutations were 481His/Asn+466Leu/Ser(92.8%,77/83). The other multiple mutations found were 481His/Asn+477Ala/Asp (6.0%,5/83), and 481His/Asn+466Leu/Ser+477Ala/Asp (1.2%,1/83). Out of 28 high-level rifampicin-resistant *S. aureus* isolates, three molecular types were found, namely, ST239-MRSA-III-*spa* t030 (25/28, 89.3%), ST239-MRSA-III-*spa* t021 (2/28, 7.1%), and ST239-MRSA-III-*spa* t045 (1/28, 3.6%).

**Conclusions:**

Rifampicin resistance in *S. aureus* was closely associated with mutations in the *rpoB* gene. High-level rifampicin-resistant *S. aureus* is one of the most important features in Anhui Provincial Hospital, and high-level rifampicin resistance in *S. aureus* is associated with multiple mutations of *rpoB* gene. The prevalence of high-level rifampicin-resistant *S. aureus* in Anhui may be associated with the spread of the ST239-MRSA III-*spa* t030 clone.

## Background

*S. aureus* is one of the most prevalent and clinically significant pathogens worldwide, which causes a variety of illnesses, ranging from minor infections of the skin to life-threatening infections with bacteremia, endocarditis, pneumonia and toxic shock syndrome [1]. With the increased use of antimicrobial agents in health care settings, multi-resistant *S. aureus* isolates have appeared and become the most common cause of nosocomial and community infections around the world [2]. Vancomycin is one of the selective drugs for MRSA infections. However, because of poor tissue diffusion and high toxicity, it is often combined with rifampicin for deep-seated infections such as osteomyelitis and endocarditis [3].

The frequency of the rifampicin-resistant (RIF-R) *S.aureus* isolates have rapidly increased. In China, the percentage of RIF-R MRSA isolates was only 15.5% in 2004 and rapidly increased to 50.2% in 2008 [4]. However, no information regarding the molecular mechanism of rifampicin resistance in *S. aureus* has been available in China. The objectives of the present study were to analyze 1) mutations in the *rpoB* gene that contributed to rifampicin resistance and 2) the molecular mechanisms of RIF-R *S. aureus* in Anhui Provincial Hospital.

## Methods

### Hospital setting

Anhui Provincial Hospital, which founded in 1898, is a major regional hospital located in the capital of Anhui Province. It is a nearly 1300-bed tertiary care teaching centre. Anhui Provincial Hospital provides healthcare services to patients from Anhui, Henan and Shandong provinces, and the average number of outpatients is about two million per year. It is also the Affiliated Hospital of Anhui Medical University and Anhui Province Medical postgraduate training base of Shandong University.

### Bacterial strains

Two hundred and eighty-three *S. aureus* were isolated from clinical specimens in the Microbiology Department of Anhui Provincial Hospital from January 2008 to December 2008. Eighty-eight RIF-R *S. aureus* isolates were re-identified by the disk diffusion method and used for the present study. The RIF-R *S. aureus* isolates represented 31% of all *S. aureus* isolates in 2008. The origin of the strains was mainly from respiratory samples and also from blood cultures, catheter-related sites, Urine samples, wound swabs, respiratory samples and exudates. Oral informed consent was given by all patients before taking the clinical specimen. The *S. aureus* isolates were re-identified by Gram’s staining, microscopic examination, coagulase testing and catalase testing. MRSA was initially screened by the cefoxitin disk diffusion method, and then confirmed by polymerase chain reaction (PCR) detecting *mecA*.

### Antimicrobial susceptibility testing

Two hundred and eighty-three *S. aureus* susceptibility to penicillin (10 units), ampicillin/sulbactam (10/10μg), cefazolin (30μg), vancomycin (30μg), erythromycin (15μg), clindamycin (2μg), rifampicin (5μg), linezolid (30μg), mupirocin (5μg), quinupristin/dalfopristin (15μg), tetracycline (30μg), trimethoprim/sulfamethoxazole (1.25/23.75μg), gentamicin (10μg), ciprofloxacin (5μg), and levofloxacin (5μg) were determined by using the disk diffusion method in accordance with standards recommended by the Clinical and Laboratory Standards Institute (CLSI) [5]. Reference strain ATCC25923 was used for quality control. MICs of rifampicin for all *S. aureus* isolates were further determined by the agar dilution method [5], and *S. aureus* ATCC 29213 and E.coli ATCC25922 were designated as RIF-S and RIF-R controls, respectively. According to the CLSI criteria [5], isolates were interpreted as RIF-S (MIC≤1 mg/L) and RIF-R (MIC≥4 mg/L) isolates.

### Detection of rifampicin resistance-associated mutations

Total DNA from *S. aureus* was purified and used as a template for amplification by PCR. An internal gene sequence of 432 bp (nucleotides 1216 to 1648), was amplified by PCR. This region included the rifampicin resistance-determining cluster I (nucleotides 1384–1464, amino acid number 462–488) and cluster II (nucleotides 1543–1590, amino acid number 515–530). The amplification was carried out in 88 RIF-R strains. Amplification was carried out as previously described [6]. The PCR products were purified and analyzed by DNA sequencing. The nucleotide sequences obtained were compared to the *rpoB* wild type sequence from *S.aureus* subsp. aureus (GenBank accession number: X64172) using the clustalw software(http://www.ebi.ac.uk/tools/clustalw/index.html).

### Molecular typing

#### SCCmec typing

SCCmec typing of MRSA isolates was performed using eight unique and specific pairs of primers for SCCmec types and subtypes I, II, III, IV and V as described previously [7].

#### Spa typing

The staphylococcus protein A (*spa*) variable repeat region from each MRSA isolate was amplified by simplex PCR oligonucleotide primers as previously described [8,9]. Purified *spa* PCR products were sequenced, and *spa* types were assigned by using the *spa* database website (http://www.ridom.de/spaserver).

#### Multilocus sequence typing (MLST)

MLST of MRSA isolates was conducted through amplification of internal fragments of seven housekeeping genes of *S.aureus* as described previously [10]. Following purification and sequencing of these genes, allele quantification and sequence typing were assigned using a well-characterized online database (http:// saureus.mlst.net/).

## Results

### Antimicrobial susceptibility patterns

Antimicrobial susceptibility testing by the disc diffusion method revealed that all RIF-R *S.aureus* isolates were MRSA and were resistant to β-lactam, ciprofloxacin, erythromycin, levofloxacin, gentamycin and tetracycline. Of the *S.aureus* isolates, 88.6% were resistant to clindamycin. Isolates also displayed low levels of resistance to sulfamethoxazole (9.1%), quinupristin (2.3%). There were no vancomycin-resistant *S.aureus* isolates in our study.

### Distribution of mutations associated with rifampicin resistance

Among the 88 RIF-R MRSA isolates, 83 isolates showed high-level rifampicin resistance (MIC ≥8 mg/L) and 5 isolates showed low-level rifampicin resistance (MICs 2 to 4 mg/L) [3,11]. Four amino acid substitutions were found in 88 RIF-R isolates. Results are shown in Table 1. Mutation at 481His/Asn was the most common and found in 95.5% of RIF-R isolates. Mutation 466Leu/Ser was found in 87.5% of isolates. The remaining mutations included 477Ala/Asp (6.8%) and 486Ser/Leu (4.5%). Five low-level resistant isolates had only one mutation, while 83 high-level resistant isolates had two or more mutations. The single mutation 481His/Asn and 486Ser/Leu were conferring low-level rifampicin resistance. Two mutations, 481His/Asn+466Leu/Ser, were the most common multiple mutations found in 92.8% (77/83) of samples. The remaining multiple mutated clones consisted of 481His/Asn+477Ala/Asp (6.0%, 5/83) and 481His/Asn+466Leu/Ser+477Ala/Asp (1.2% and 1/83, respectively).

**Table 1:** The characteristics of the rifampicin-resistant S. aureus isolates studied

**MRSA rpoB mutations**		**Number of isolates**	**Mutation frequency %**	**Rifampicin MIC**		**Resistance pattern**
**Nucleotide mutation**	**Amino acid substitution**			**MIC(mg/L)**	**Number of isolates**	
TCA/TTA	486Leu/Ser	4	4.5%	4	4	CIP+E+GEN+TET(3)
						CIP+E+GEN+TET+CC (1)
CAT/AAT	481His/Asn	1	1.1%	4	1	CIP+E+GEN+TET(1)
CAT/AAT+TTA/TCA	481His/Asn+466Leu/Ser	45	87.5%	32	45	CIP+E+GEN+TET(7)
						CIP+E+GEN+TET+CC (35)
						CIP+E+GEN+TET+CC+SXT(2)
						CIP+E+GEN+TET+CC+SXT+QD(1)
CAT/AAT+TTA/TCA	481His/Asn+466Leu/Ser	14		64	14	CIP+E+GEN+TET+CC (12)
						CIP+E+GEN+TET+CC +SXT(2)
CAT/AAT+TTA/TCA	481His/Asn+466Leu/Ser	11		128	11	CIP+E+GEN+TET+CC (8)
						CIP+E+GEN+TET+CC +SXT(3)
CAT/AAT+TTA/TCA	481His/Asn+466Leu/Ser	7		256	7	CIP+E+GEN+TET+CC +SXT(7)
CAT/AAT+GCT/GAT	481His/Asn+477Ala/Asp	5	5.7%	64	5	CIP+E+GEN+TET+CC (5)
CAT/AAT+TTA/TCA+GCT/GAT	481His/Asn+466Leu/Ser+477Ala/Asp	1	1.1%	128	1	CIP+E+GEN+TET+CC (1)

### Molecular typing

SCC*mec* typing, MLST and *spa* typing were carried out in the 28 high-level RIF-R MRSA strains. Results are shown in Table 2. The majority (n =25, 89.3%) belonged to a common molecular type, ST239-MRSAIII-*spa* t030. The remaining molecular types were identified as ST239-MRSA-III-*spa* t021 (2/28, 7.1%) and ST239-MRSA-III-*spa* t045 (1/28, 3.6%).

**Table 2:** **Molecular features of 28 high-level rifampicin-resistant *S. aureus *isolates**

**MLST (ST)**	**SCCmec type**	**spa-type**	**Number of isolates**	**Nucleotide mutation**	**Amino acid substitution**	**Resistance pattern**
ST239	III	t030	24	CAT/AAT+TTA/TCA	481His/Asn+466Leu/Ser	CIP+E+GEN+TET(1)
						CIP+E+GEN+TET+CC(23)
			1	CAT/AAT+GCT/GAT	481His/Asn+477Ala/Asp	CIP+E+GEN+TET+CC (1)
ST239	III	t021	2	CAT/AAT+TTA/TCA	481His/Asn+466Leu/Ser	CIP+E+GEN+TET+CC(2)
ST239	III	t045	1	CAT/AAT+TTA/TCA	481His/Asn+466Leu/Ser	CIP+E+GEN+TET+CC+SXT(1)

CIP, ciprofloxacin; E, erythromycin; CC, clindamycin; TET, tetracycline; SXT, sulfamethoxazole/trimethoprim; GEN, gentamycin; QD, quinupristin/dalfopristin.

## Discussion

Multiresistance and high infection rates are common features of *S .aureus* and are growing problems in hospital settings. The high prevalence of antibiotic resistance in *S. aureus* nosocomial isolates is currently explained by intensive use of topical and systemic antimicrobial agents in health care settings, which represents a highly selective pressure for antibiotic-resistant bacterial clones [12]. In particular, MRSA strains showed high resistance rates to various antibiotics [13]. The proportion of MRSA isolates has increased in recent years. In China, surveillance data of bacterial resistance in 1998–1999 showed that the percentage of MRSA was 37.4% [14] and rapidly reached 51.7% in 2010 [4].

Rifampicin is an antibiotic of significant interest in the rise of MRSA infections. A combination therapy, with an antibiotic such as vancomycin often is required to reach deep-seated infections effectively. Rifampicin acts by interacting specifically with bacterial RNA polymerase encoded by the gene *rpoB*[15]. Rifampicin resistance emerges easily in *S. aureus*, in particular in methicillin-resistant Strains [3].The prevalence of RIF-R MRSA has risen rapidly in the past few years and remains at a high resistance rate. In China, the data obtained from the surveillance of bacterial resistance showed that the percentage of RIF-R MRSA was 15.5% in 2004 and rapidly reached 49.6% by 2006. The percentage remained high from 2006 to 2009 [4]. Obviously, the nature of RIF-R MRSA isolates represents a therapeutic challenge for treating serious MRSA infections. Most RIF-R MRSA isolates were high-level resistant in our study and the percentage was found to be 94.3%. In fact, it was higher than the rate reported in some European countries, such as Spain, which had a rate of 3.7% (4/108) in 2010 [6]. There were two reasons that could explain the difference between the Rif-R rate in China compared to other countries. One possibility was the intensive use of some antimicrobial agents in health care settings in China such as quinolones, which not only could lead to the selection of multi-resistant nosocomial isolates of *S. aureus*, but also potentially induce endogenous, resistance-conferring mutations in bacterial genes that encode drug targets. A second possibility might be that the prevalence of MRSA clones in China was different from European countries.

For a variety of bacteria, such as E. coli [16], Mycobacterium tuberculosis [17] and *S .aureus*[3], the main mutations responsible for rifampicin resistance were in a particular region encompassing a few hundred nucleotides called the rifampicin resistance-determining region (RRDR). In *S. aureus* the RRDR was divided into two clusters which were designated cluster I (nucleotides 1384–1464, amino acids 462–488) and cluster II (nucleotides 1543–1590, amino acids 515–530). As described in previous studies, the two clusters were also both closely associated with rifampicin resistance [3,18].

Here, we have amplified and sequenced portions of *rpoB* from RIF-R *S.aureus* isolates. All four amino acid substitutions we identified were present in cluster I. Mutation 481His/Asn was the most prevalent one. The majority (n = 84, 96%) of the 88 RIF-R MRSA isolates harbored the amino acid substitution 481His/Asn, which was in line with previous reports [3,19]. Our results further confirm that 481His/Asn has a major impact on the occurrence and development of rifampicin resistance in *S. aureus*. High-level rifampicin resistance may also be attributed to additional mutations within *rpoB*, as previously described [20]. The additional mutations we found were 466Leu/Ser and 477Ala/Asp. Isolates containing multiple mutations, 481His/Asn and 466Leu/Ser,were reported by other studies, which also showed high-level rifampicin resistance [18,19]. Mutational changes at amino acid position 477 have also been reported by several groups [3,6,18], but the mutation rate was low and the types of amino acid substitutions which arose were different.

MRSA infections have been caused by a relatively small number of epidemic MRSA clones. As described in previous studies, the two major epidemic MRSA clones identified in China from 2005 to 2006 were ST239-MRSA III and ST5-MRSA II [21]. A pandemic MRSA clone ST239, which was found to be derived from ST8 and ST30 parental strains through simple chromosome replacement instead of movement of mobile genetic elements, was first found in Brazil and widely spread throughout the world [22]. In Asia and in China, ST239 accounted for 97% of nosocomial MRSA infections [23]. ST239-MRSA III was also the major clone found in our study. Staphylococcal protein A (*SpA*) is a cell wall anchored virulence factor [24]. Our research shows that most strains with RIF-R *S. aureus* belong to ST239-MRSAIII-*spa* t030, a situation in accordance with Chen et al. [25]. Their research showed t030 was up to 89.6% of MRSA in Peking Union Medical College Hospital (PUMCH) in 2002. In addition, t030 was also found to be rifampicin resistant by Chen et al., which was the main difference with t037. Our results are in line with these reports. These findings indicate that ST239-MRSAIII-*spa* t030 strains, associated with high-level rifampicin resistance, have spread in Anhui Provincial Hospital. Therefore, bacterial resistance surveillance and the control of hospital infections should take these findings into consideration in order to prevent and limit the spread of high-level rifampicin resistant *S. aureus.*

## Conclusion

Most RIF-R MRSA isolates were high-level resistant in our study. Rifampicin-resistance in *S .aureus* is closely associated with mutations which occur in the *rpoB* gene. ST239- MRSA III-*spa* t030 strains, which was associated with the high-level rifampicin resistance, has spread in Anhui Provincial Hospital.

## Competing interests

We have no any Competing interests. Our manuscript doesn’t involve any ethical issues.

## Authors' contributions

WZ, XM conceived the study and participated in its design. YD, HL participated in field and clinical aspects of the study. WZ carried out laboratory work. WS, WZ drafted the manuscript. XM, WS, WZ, XZ, WC edited the manuscript. All authors read and approved the final version of the manuscript.

## References

[B1] Lowy FD Staphylococcus aureus infections N Engl J Med 1998 339 8 520 532 10.1056/NEJM199808203390806 9709046

[B2] Deresinski S Methicillin-resistant Staphylococcus aureus: an evolutionary, epidemiologic, and therapeutic odyssey Clin Infect Dis 2005 40 4 562 573 10.1086/427701 15712079

[B3] Aubry-Damon H Soussy CJ Courvalin P Characterization of mutations in the rpoB gene that confer rifampin resistance in Staphylococcus aureus Antimicrob Agents Chemother 1998 42 10 2590 2594 9756760 10.1128/aac.42.10.2590 PMC105902

[B4] Xiao YH Giske CG Wei ZQ Shen P Heddini A Li LJ Epidemiology and characteristics of antimicrobial resistance in China Drug Resist Updat 2011 14 4–5 236 250 21807550 10.1016/j.drup.2011.07.001

[B5] Hindler J The 2008 CLSI Standard for Antimicrobial Susceptibiltiy Testing 2008 Jan: APHL Teleconference

[B6] Mick V Dominguez MA Tubau F Linares J Pujol M Martin R Molecular characterization of resistance to Rifampicin in an emerging hospital-associated Methicillin-resistant Staphylococcus aureus clone ST228 Spain. BMC Microbiol 2010 10 68 10.1186/1471-2180-10-68 PMC2844403 20202188

[B7] Zhang K McClure JA Elsayed S Louie T Conly JM Novel multiplex PCR assay for characterization and concomitant subtyping of staphylococcal cassette chromosome mec types I to V in methicillin-resistant Staphylococcus aureus J Clin Microbiol 2005 43 10 5026 5033 10.1128/JCM.43.10.5026-5033.2005 16207957 PMC1248471

[B8] Koreen L Ramaswamy SV Graviss EA Naidich S Musser JM Kreiswirth BN spa typing method for discriminating among Staphylococcus aureus isolates: implications for use of a single marker to detect genetic micro- and macrovariation J Clin Microbiol 2004 42 2 792 799 10.1128/JCM.42.2.792-799.2004 14766855 PMC344479

[B9] Harmsen D Claus H Witte W Rothganger J Turnwald D Vogel U Typing of methicillin-resistant Staphylococcus aureus in a university hospital setting by using novel software for spa repeat determination and database management J Clin Microbiol 2003 41 12 5442 5448 10.1128/JCM.41.12.5442-5448.2003 14662923 PMC309029

[B10] Enright MC Day NP Davies CE Peacock SJ Spratt BG Multilocus sequence typing for characterization of methicillin-resistant and methicillin-susceptible clones of Staphylococcus aureus J Clin Microbiol 2000 38 3 1008 1015 10698988 10.1128/jcm.38.3.1008-1015.2000 PMC86325

[B11] Wichelhaus TA Boddinghaus B Besier S Schafer V Brade V Ludwig A Biological cost of rifampin resistance from the perspective of Staphylococcus aureus Antimicrob Agents Chemother 2002 46 11 3381 3385 10.1128/AAC.46.11.3381-3385.2002 12384339 PMC128759

[B12] Didier JP Villet R Huggler E Lew DP Hooper DC Kelley WL Vaudaux P Impact of ciprofloxacin exposure on Staphylococcus aureus genomic alterations linked with emergence of rifampin resistance Antimicrob Agents Chemother 2011 55 5 1946 1952 10.1128/AAC.01407-10 21357297 PMC3088276

[B13] Chen R Yan ZQ Feng D Luo YP Wang LL Shen DX Nosocomial bloodstream infection in patients caused by Staphylococcus aureus: drug susceptibility, outcome, and risk factors for hospital mortality Chin Med J (Engl) 2012 125 2 226 229 22340550

[B14] Li J Weinstein AJ Yang M [Surveillance of bacterial resistance in China (1998–1999)] Zhonghua Yi Xue Za Zhi 2001 81 1 8 16 11798843

[B15] Campbell EA Korzheva N Mustaev A Murakami K Nair S Goldfarb A Darst SA Structural mechanism for rifampicin inhibition of bacterial rna polymerase Cell 2001 104 6 901 912 10.1016/S0092-8674(01)00286-0 11290327

[B16] Jin DJ Gross CA Mapping and sequencing of mutations in the Escherichia coli rpoB gene that lead to rifampicin resistance J Mol Biol 1988 202 1 45 58 10.1016/0022-2836(88)90517-7 3050121

[B17] Bolotin S Alexander DC Chedore P Drews SJ Jamieson F Molecular characterization of drug-resistant Mycobacterium tuberculosis isolates from Ontario, Canada J Antimicrob Chemother 2009 64 2 263 266 10.1093/jac/dkp183 19520719

[B18] Wichelhaus TA Schafer V Brade V Boddinghaus B Molecular characterization of rpoB mutations conferring cross-resistance to rifamycins on methicillin-resistant Staphylococcus aureus Antimicrob Agents Chemother 1999 43 11 2813 2816 10543773 10.1128/aac.43.11.2813 PMC89569

[B19] O'Neill AJ Huovinen T Fishwick CW Chopra I Molecular genetic and structural modeling studies of Staphylococcus aureus RNA polymerase and the fitness of rifampin resistance genotypes in relation to clinical prevalence Antimicrob Agents Chemother 2006 50 1 298 309 10.1128/AAC.50.1.298-309.2006 16377701 PMC1346782

[B20] Frenay HM Bunschoten AE Schouls LM van Leeuwen WJ Vandenbroucke-Grauls CM Verhoef J Mooi FR Molecular typing of methicillin-resistant Staphylococcus aureus on the basis of protein A gene polymorphism Eur J Clin Microbiol Infect Dis 1996 15 1 60 64 10.1007/BF01586186 8641305

[B21] Liu Y Wang H Du N Shen E Chen H Niu J Ye H Chen M Molecular evidence for spread of two major methicillin-resistant Staphylococcus aureus clones with a unique geographic distribution in Chinese hospitals Antimicrob Agents Chemother 2009 53 2 512 518 10.1128/AAC.00804-08 19029328 PMC2630620

[B22] Harris SR Feil EJ Holden MT Quail MA Nickerson EK Chantratita N Gardete S Tavares A Day N Lindsay JA Evolution of MRSA during hospital transmission and intercontinental spread Science 2010 327 5964 469 474 10.1126/science.1182395 20093474 PMC2821690

[B23] Ma XL Chen FH Zhou X Chang WJ Dai YY Molecular characteristic of Staphylococcus aureus isolates in a Chinese teaching hospital African Journal of Microbiology Research 2011 5 19 2969 2974

[B24] Coombs GW Monecke S Ehricht R Slickers P Pearson JC Tan HL Christiansen KJ O'Brien FG Differentiation of clonal complex 59 community-associated methicillin-resistant Staphylococcus aureus in Western Australia Antimicrob Agents Chemother 2010 54 5 1914 1921 10.1128/AAC.01287-09 20211891 PMC2863625

[B25] Chen H Liu Y Jiang X Chen M Wang H Rapid change of methicillin-resistant Staphylococcus aureus clones in a Chinese tertiary care hospital over a 15-year period Antimicrob Agents Chemother 2010 54 5 1842 1847 10.1128/AAC.01563-09 20176895 PMC2863666

